# Rényi Cross-Entropy Measures for Common Distributions and Processes with Memory

**DOI:** 10.3390/e24101417

**Published:** 2022-10-04

**Authors:** Ferenc Cole Thierrin, Fady Alajaji, Tamás Linder

**Affiliations:** Department of Mathematics and Statistics, Queen’s University, Kingston, ON K7L 3N6, Canada

**Keywords:** Rényi information measures, cross-entropy, divergence measures, exponential family distributions, Gaussian processes, Markov sources

## Abstract

Two Rényi-type generalizations of the Shannon cross-entropy, the Rényi cross-entropy and the Natural Rényi cross-entropy, were recently used as loss functions for the improved design of deep learning generative adversarial networks. In this work, we derive the Rényi and Natural Rényi differential cross-entropy measures in closed form for a wide class of common continuous distributions belonging to the exponential family, and we tabulate the results for ease of reference. We also summarise the Rényi-type cross-entropy rates between stationary Gaussian processes and between finite-alphabet time-invariant Markov sources.

## 1. Introduction

The Rényi entropy [1] of order α of a probability mass function *p* with finite support S is defined as

(1) $$H_{\alpha }\left(p\right)=\frac{1}{1-\alpha }\ln\sum_{x\in S}p{\left(x\right)}^{\alpha }$$

for α>0,α≠1. The Rényi entropy generalizes the Shannon entropy,

(2) $$H\left(p\right)=-\sum_{x\in S}p\left(x\right)\ln p\left(x\right),$$

in the sense that as α→1, $H_{\alpha }\left(p\right)\to H\left(p\right)$. Several other Rényi-type information measures have been put forward, each obeying the condition that their limit as α goes to one reduces to a Shannon-type information measure. This includes the Rényi divergence (of order α) between two discrete distributions *p* and *q* with common finite support S, given by

(3) $$D_{\alpha }\left(p||q\right)=\frac{1}{\alpha -1}\ln\sum_{x\in S}p{\left(x\right)}^{\alpha }q{\left(x\right)}^{1-\alpha }$$

which reduces to the familiar Kullback–Leibler divergence,

(4) $$D_{\mathrm{KL}}\left(p||q\right)=\sum_{x\in S}p\left(x\right)\ln\frac{p(x)}{q(x)},$$

as α→1. Note that in some cases [2], there may exist multiple Rényi-type generalisations for the same information measure (particularly for mutual information).

Many of these definitions admit natural counterparts in the case when the involved distributions have a probability density function (pdf). This gives rise to information measures such as the Rényi differential entropy for pdf *p* with support S,

(5) $$h_{\alpha }\left(p\right)=\frac{1}{1-\alpha }\ln\int_{S}p{\left(x\right)}^{\alpha }\;dx,$$

and the Rényi differential divergence between pdfs *p* and *q* with common support S,

(6) $$D_{\alpha }\left(p∥q\right)=\frac{1}{\alpha -1}\ln\int_{S}p{\left(x\right)}^{\alpha }q{\left(x\right)}^{1-\alpha }\;dx.$$

The Rényi cross-entropy between distributions *p* and *q* is an analogous generalization of the Shannon cross-entropy

(7) $$H\left(p;q\right)=-\sum_{x\in S}p\left(x\right)\ln q\left(x\right).$$

Two definitions for this measure have been recently suggested. In light of the fact that Shannon’s cross-entropy satisfies H(p;q)=D(p∥q)+H(p), a natural definition of the Rényi cross-entropy is:

(8) $${\tilde{H}}_{\alpha }\left(p;q\right)D_{\alpha }\left(p||q\right)+H_{\alpha }\left(p\right).$$

This definition was indeed proposed in [3] in the continuous case, with the differential cross-entropy measure given by

(9) $${\tilde{h}}_{\alpha }\left(p;q\right)D_{\alpha }\left(p||q\right)+h_{\alpha }\left(p\right).$$

In contrast, prior to [3], the authors of [4] introduced the Rényi cross-entropy in their study of shifted Rényi measures expressed as the logarithm of weighted generalized power means. Specifically, upon simplifying Definition 6 in [4], their expression for the Rényi cross-entropy between distributions *p* and *q* is given by

(10) $$H_{\alpha }\left(p;q\right)\frac{1}{1-\alpha }\ln\sum_{x\in S}p\left(x\right)q{\left(x\right)}^{\alpha -1}.$$

For the continuous case, (10) can be readily converted to yield the Rényi differential cross-entropy between pdfs *p* and *q*:

(11) $$h_{\alpha }\left(p;q\right)\frac{1}{1-\alpha }\ln\int_{S}p\left(x\right)q{\left(x\right)}^{\alpha -1}\;dx.$$

Note that both (8) and (10) reduce to the Shannon cross-entropy H(p;q) as α→1 [5]. A similar result holds for (9) and (11), where the Shannon differential cross-entropy,

(12) $$h\left(p;q\right)=-\int_{S}p\left(x\right)\ln q\left(x\right)\;dx,$$

is obtained. Further, the Rényi (differential) entropy is recovered in all equations when p=q (almost everywhere). These properties alone make these definitions viable extensions of the Shannon (differential) cross-entropy.

Finding closed-form expressions for the cross-entropy measure in (9) for continuous distributions is direct, since the Rényi divergence and the Rényi differential entropy were already calculated for numerous distributions in [6,7], respectively. However, deriving the measure in (11) is more involved. We hereafter refer to the measures ${\tilde{H}}_{\alpha }\left(p;q\right)$ in (8) and ${\tilde{h}}_{\alpha }\left(p;q\right)$ in (9) as the *Natural Rényi cross-entropy* and the *Natural Rényi differential cross-entropy*, respectively; while we plainly call the measures $H_{\alpha }\left(p;q\right)$ in (10) and $h_{\alpha }\left(p;q\right)$ (11) as the *Rényi cross-entropy* and the *Rényi differential cross-entropy*, respectively.

In a recent conference paper [8], we showed how to calculate the Rényi differential cross-entropy $h_{\alpha }\left(p;q\right)$ between distributions of the same type from the exponential family. Building upon the results shown there, the purpose of this paper is to derive in closed form the expression of $h_{\alpha }\left(p;q\right)$ for thirteen commonly used univariate distributions from the exponential family, as well as for multivariate Gaussians, and tabulate the results for ease of reference. We also analytically derive the Natural Rényi differential cross-entropy ${\tilde{h}}_{\alpha }\left(p;q\right)$ for the same set of distributions. Finally, we present tables summarising the Rényi and Natural Rényi (differential) cross-entropy rate measures, along with their Shannon counterparts, for two important classes of sources with memory, namely stationary Gaussian sources and finite-state time-invariant Markov sources.

Motivation for determining formulae for the Rényi cross-entropy originates from the use of the Shannon differential cross-entropy as a loss function for the design of deep learning generative adversarial networks (GANs) in [9]. The parameter α, ubiquitous to all Rényi information measures, allows one to fine-tune the loss function to improve the quality of the GAN-generated output. This can be seen in [3,5,10], which used the Rényi differential cross-entropy, and the Natural Rényi differential cross-entropy measures, respectively, to generalize the original GAN loss function (which is recovered as α→1), resulting in both improved GAN system stability and performance for multiple image datasets. It is also shown in [5,10] that the introduced Rényi-centric generalized loss function preserves the equilibrium point satisfied by the original GAN via the so-called Jensen-Rényi divergence [11], a natural extension of the Jensen–Shannon divergence [12] upon which the equilibrium result of [9] is established. Other GAN systems that utilize different generalized loss functions were recently developed and analysed in [13,14,15] (see also the references therein for prior work).

The rest of this paper is organised as follows. In Section 2, the formulae for the Rényi differential cross-entropy and Natural Rényi differential cross-entropy for distributions from the exponential family are given. In Section 3, these calculations are systematically carried out for fourteen pairs of distributions of the same type within the exponential family, and the results are presented in two tables. The Rényi and Natural Rényi differential cross-entropy rates are presented in Section 4 for stationary Gaussian sources; furthermore, the Rényi and Natural Rényi cross-entropy rates are provided in Section 5 for finite-state time-invariant Markov sources. Finally, the paper is concluded in Section 6.

## 2. Rényi and Natural Rényi Differential Cross-Entropies for Distributions from the Exponential Family

An exponential family is a class of probability distributions over a support $S\subseteq R^{n}$ defined by a parameter space $\Theta \subseteq R^{m}$ and functions b:S↦R, c:Θ↦R, $T:S\mapsto R^{m}$, and $\eta :\Theta \mapsto R^{m}$ such that the pdf in this family has the form

(13) $$f\left(x\right)=c\left(\theta \right)b\left(x\right)\exp\left(\langle \eta (\theta ),T(x)\rangle \right),\;x\in S$$

where 〈·,·〉 denotes the standard inner product in $R^{m}$. Alternatively, by using the (natural) parameter η=η(θ), the pdf can also be written

(14) $$f\left(x\right)=b\left(x\right)\exp\left(\langle \eta ,T(x)\rangle +A(\eta )\right),$$

where A(η):η(Θ)↦R with A(η)=lnc(θ). Examples of important pdfs we consider from the exponential family are included in Appendix A.

In [8], the cross-entropy between pdfs $f_{1}$ and $f_{2}$ of the same type from the exponential family was proven to be

(15) $$h_{\alpha }\left(f_{1};f_{2}\right)=\frac{A\left(\eta_{1}\right)-A\left(\eta_{h}\right)+\ln E_{h}}{1-\alpha }-A\left(\eta_{2}\right),$$

where

(16) $$E_{h}=E_{f_{h}}\left[b{\left(X\right)}^{\alpha -1}\right]=\int b{\left(x\right)}^{\alpha -1}f_{h}\left(x\right)\;dx.$$

Here, $f_{h}$ refers to a distribution of the same type as $f_{1}$ and $f_{2}$ within the exponential family with natural parameter

(17) $$\eta_{h}\eta_{1}+\left(\alpha -1\right)\eta_{2}.$$

It can also be shown that the Natural Rényi differential cross-entropy between $f_{1}$ and $f_{2}$ is given by

(18) $${\tilde{h}}_{\alpha }\left(f_{1};f_{2}\right)=\frac{A\left(\eta_{\alpha }\right)-A\left(\alpha \eta_{1}\right)+\ln E_{\alpha }}{1-\alpha }-A\left(\eta_{2}\right),$$

where

(19) $$\eta_{\alpha }=\alpha \eta_{1}+\left(1-\alpha \right)\eta_{2},$$

and

(20) $$E_{\alpha }=E_{f_{\alpha 1}}\left[b{\left(X\right)}^{\alpha -1}\right]=\int b{\left(x\right)}^{\alpha -1}f_{\alpha 1}\left(x\right)\;dx$$

where $f_{\alpha 1}$ refers to a distribution of the same type as $f_{1}$ and $f_{2}$ within the exponential family with natural parameter $\alpha \eta_{1}$.

## 3. Tables of Rényi and Natural Rényi Differential Cross-Entropies

Table 1 and Table 2 list Rényi and Natural differential cross-entropy expressions, respectively, between common distributions of the same type from the exponential family (which we describe in Appendix A for convenience). The closed-form expressions were derived using (15) and (18), respectively. In the tables, the subscript *i* is used to denote that a parameter belongs to pdf $f_{i}$, i=1,2.

## 4. Rényi and Natural Rényi Differential Cross-Entropy Rates for Stationary Gaussian Processes

In [8], the Rényi differential cross-entropy rate between two stationary zero-mean Gaussian processes is derived. We present its formula alongside the expressions of the Shannon and Natural Rényi differential cross-entropy rates in Table 3. Here, f(λ) is the spectral density of the first zero-mean Gaussian process, g(λ) is the spectral density of the second Gaussian process,

(21) h(λ)=f(λ)+(α−1)g(λ),

and

(22) j(λ)=αf(λ)+(1−α)g(λ).

## 5. Rényi and Natural Rényi Cross-Entropy Rates for Markov Sources

In [8], the Rényi cross-entropy rate between finite-state time-invariant Markov sources was established, using, as in [16], tools from the theory of non-negative matrices and Perron–Frobenius theory (e.g., cf. [17,18]). This measure, as well as the Shannon and Natural Rényi differential cross-entropy rates, are derived and summarised in Table 4. Here, *P* and *Q* are the m×m (stochastic) transition matrices associated with the first and second Markov sources, respectively, where both sources have a common alphabet of size *m*. To allow any value of the Rényi parameter α in (0,1)∪(1,∞), we assume that the transition matrix *Q* of the second Markov chain has positive entries (Q>0); however, the transition matrix *P* of the first Markov chain is taken to be an *arbitrary* stochastic matrix. For simplicity, we assume that the initial distribution vectors, *p* and *q*, of both Markov chains also have positive entries (p>0 and q>0). This condition can be relaxed via the approach used to prove Theorem 1 in [16]. Moreover, $\pi_{p}^{T}$ denotes the stationary probability row vector associated with the first Markov chain, and 1 is an *m*-dimensional column vector in which each element equals one. Furthermore, ⊙ denotes element-wise multiplication (i.e., the Hadamard product operation), and $\dot{\ln}$ is the element-wise natural logarithm.

Finally, the definition of $\lambda \left(R\right):R^{m\times m}\mapsto R$ for a matrix *R* is more involved. If *R* is irreducible, λ(R) is its largest positive eigenvalue. Otherwise, rewriting *R* in its canonical form as detailed in Proposition 1 in [16], we have that $\lambda \left(R\right)=\max\left(\lambda^{*},\lambda_{*}\right)$, where $\lambda^{*}$ is the maximum of all largest positive eigenvalues of (irreducible) sub-matrices of *R* corresponding to self-communicating classes, and $\lambda_{*}$ is the maximum of all largest positive eigenvalues of sub-matrices of *R* corresponding to classes reachable from an inessential class.

## 6. Conclusions

We have derived closed-form formulae for the Rényi and Natural Rényi differential cross-entropies of commonly used distributions from the exponential family. This is of potential use to further studies in information theory and machine learning, particularly problems where deep neural networks, trained according to a Shannon cross-entropy loss function, can be improved via generalized Rényi-type loss functions in virtue of the extra degree of freedom provided by the Rényi (α) parameter. In addition, we have provided formulae for the Rényi and Natural Rényi differential cross-entropy rates for stationary zero-mean Gaussian processes and expressions for the cross-entropy rates for Markov sources. Further work includes expanding the present collection by considering distributions such as Levy or Weibull and investigating cross-entropy measures based on the *f*-divergence [19,20,21], starting with Arimoto’s divergence [22].

## Figures and Tables

**Table 1:** Rényi differential cross-entropies.

Name	$h_{\alpha }\left(f_{1};f_{2}\right)$
**Beta**	$\ln B(a_{2},b_{2})+\frac{1}{\alpha -1}\ln\frac{B(a_{h},b_{h})}{B(a_{1},b_{1})}$
	$a_{h}:=a_{1}+\left(\alpha -1\right)\left(a_{2}-1\right)$,$\;a_{h}>0$
	$b_{h}:=b_{1}+\left(\alpha -1\right)\left(b_{2}-1\right)$,$\;b_{h}>0$
$\begin{array}{c} \chi \\ \left(\mathrm{scaled}\right) \end{array}$	$\frac{1}{2}\left(k_{2}\ln\sigma_{2}^{2}\sigma_{h}^{2}-\ln2\sigma_{h}^{2}\right)+\ln\Gamma \left(\frac{k_{2}}{2}\right)$
	$+\frac{1}{\alpha -1}\left(\ln\Gamma \left(\frac{k_{h}}{2}\right)-\ln\Gamma \left(\frac{k_{1}}{2}\right)-\frac{k_{1}}{2}\ln\sigma_{1}^{2}\sigma_{h}^{2}\right)$
	$\sigma_{h}^{2}:=\frac{1}{\sigma_{1}^{2}}+\frac{\alpha -1}{\sigma_{2}^{2}}$,$\;\sigma_{h}^{2}>0$
	$k_{h}:=k_{1}+\left(\alpha -1\right)\left(k_{2}-1\right)$,$\;k_{h}>0$
$\begin{array}{c} \chi \\ \left(\mathrm{non-scaled}\right) \end{array}$	$\frac{1}{2}\left(k_{2}\ln\alpha -\ln2\alpha \right)+\ln\Gamma \left(\frac{k_{2}}{2}\right)$
	$+\frac{1}{\alpha -1}\left(\ln\Gamma \left(\frac{k_{h}}{2}\right)-\ln\Gamma \left(\frac{k_{1}}{2}\right)-\frac{k_{1}}{2}\ln\alpha \right)$
	$k_{h}:=k_{1}+\left(\alpha -1\right)\left(k_{2}-1\right)$,$\;k_{h}>0$
$\chi^{2}$	$\frac{1}{1-\alpha }\left(\frac{\nu_{1}}{2}\ln\left(\alpha \right)-\ln\Gamma \left(\frac{\nu_{1}}{2}\right)+\ln\Gamma \left(\frac{\nu_{h}}{2}\right)\right)$
	$+\frac{2-\nu_{2}}{2}\ln\left(\alpha \right)+\ln2\Gamma \left(\frac{\nu_{2}}{2}\right)$
	$\nu_{h}:=\nu_{1}+\left(\alpha -1\right)\left(\nu_{2}-2\right)$,$\;\nu_{h}>0$
**Exponential**	$\frac{1}{1-\alpha }\ln\frac{\lambda_{1}}{\lambda_{h}}-\ln\lambda_{2}$
	$\lambda_{h}:=\lambda_{1}+\left(\alpha -1\right)\lambda_{2}$,$\;\lambda_{h}>0$
**Gamma**	$\ln\Gamma \left(k_{2}\right)+k_{2}\ln\theta_{2}$
	$+\frac{1}{1-\alpha }\left(\ln\frac{\Gamma (k_{h})}{\Gamma (k_{1})}-k_{h}\ln\theta_{h}-k_{1}\ln\theta_{1}\right)$
	$\theta_{h}:=\frac{\theta_{1}+\left(a-1\right)\theta_{2}}{\left(\alpha -1\right)\theta_{1}\theta_{1}}$,$\;\theta_{h}>0$
	$k_{h}:=k_{1}+\left(\alpha -1\right)k_{2}$,$\;k_{h}>0$
$\begin{array}{c} \mathrm{Gaussian}\\ \left(\mathrm{univariate}\right) \end{array}$	$\frac{1}{2}\left(\ln\left(2\pi \sigma_{2}^{2}\right)+\frac{1}{1-\alpha }\ln\left(\frac{\sigma_{2}^{2}}{{\left(\sigma^{2}\right)}_{h}}\right)+\frac{{\left(\mu_{1}-\mu_{2}\right)}^{2}}{{\left(\sigma^{2}\right)}_{h}}\right)$
	${\left(\sigma^{2}\right)}_{h}:=\sigma_{2}^{2}+\left(\alpha -1\right)\sigma_{1}^{2}$,$\;{\left(\sigma^{2}\right)}_{h}>0$
$\begin{array}{c} \mathrm{Gaussian}\\ \left(\mathrm{multivariate}\right) \end{array}$	$\frac{1}{2-2\alpha }\left(-\ln|A||\Sigma_{1}|+\left(1-\alpha \right)\ln{\left(2\pi \right)}^{n}\left|\Sigma_{2}\right|-d\right)$
	$A:=\Sigma_{1}^{-1}+\left(\alpha -1\right)\Sigma_{2}^{-1}$, A≻0
	$d:=\mu_{1}^{T}\Sigma_{1}^{-1}\mu_{1}+\left(\alpha -1\right)\mu_{2}^{T}\Sigma_{2}^{-1}\mu_{2}$
	$-\left(\mu_{1}^{T}\Sigma_{1}^{-1}+\left(\alpha -1\right)\mu_{2}^{T}\Sigma_{2}^{-1}\right)A^{-1}\left(\Sigma_{1}^{-1}\mu_{1}+\left(\alpha -1\right)\Sigma_{2}^{-1}\mu_{2}\right)$
$\begin{array}{c} \mathrm{Gumbel}\\ (\beta_{1}=\beta_{2}=\beta )\; \end{array}$	$\frac{1}{1-\alpha }\left(\ln\frac{\Gamma (2-\alpha )}{\beta }-\frac{\mu_{1}}{\beta }-\alpha \ln\eta_{h}\right)+\frac{\mu_{2}}{\beta }$
	$\eta_{h}:=e^{-\mu_{1}/\beta }+\left(\alpha -1\right)e^{-\mu_{2}/\beta }$,$\;\eta_{h}>0$
**Half-Normal**	$\frac{1}{2}\left(\ln\left(\frac{\pi \sigma_{2}^{2}}{2}\right)+\frac{1}{1-\alpha }\ln\left(\frac{\sigma_{2}^{2}}{{\left(\sigma^{2}\right)}_{h}}\right)\right)$
	${\left(\sigma^{2}\right)}_{h}:=\sigma_{2}^{2}+\left(\alpha -1\right)\sigma_{1}^{2}$,$\;{\left(\sigma^{2}\right)}_{h}>0$
$\begin{array}{c} \mathrm{Laplace}\\ \left(\mu_{1}=\mu_{2}=0\right) \end{array}$	$\ln\left(2b_{2}\right)+\frac{1}{1-\alpha }\ln\left(\frac{b_{2}}{2b_{h}}\right)$
	$b_{h}:=b_{2}+\left(1-\alpha \right)b_{1}$,$\;b_{h}>0$
$\begin{array}{c} \mathrm{Maxwell}\\ \mathrm{Boltzmann} \end{array}$	$\frac{1}{2}\left(\ln2\pi +3\ln\sigma_{2}^{2}\right)+\ln\sigma_{h}^{2}$
	$+\frac{1}{1-\alpha }\left(\ln\frac{\Gamma (2\alpha )}{\Gamma (\alpha )}-\frac{3}{2}\ln\sigma_{1}^{2}\sigma_{h}^{2}\right)$
	$\sigma_{h}^{2}:=\sigma_{1}^{-2}+\left(\alpha -1\right)\sigma_{2}^{-2}$,$\;\sigma_{h}^{2}>0$
$\begin{array}{c} \mathrm{Pareto}\\ \left(m_{1}=m_{2}=m\right) \end{array}$	$-\ln m-\ln\lambda_{2}+\frac{1}{1-\alpha }\ln\frac{\lambda_{1}}{\lambda_{h}}$
	$\lambda_{h}:=\lambda_{1}+\left(\alpha -1\right)\left(\lambda_{2}+1\right)$,$\;\lambda_{h}>0$
**Rayleigh**	$\frac{\ln\sigma_{1}^{2}-\alpha \ln\sigma_{h}^{2}+\ln\Gamma \left(\frac{1-\alpha }{2}\right)}{1-\alpha }+\ln2\sigma_{2}^{2}$
	$\sigma_{h}^{2}:=\sigma_{1}^{-2}+\left(\alpha -1\right)\sigma_{2}^{-2}$,$\;\sigma_{h}^{2}>0$

**Table 2:** Natural Rényi differential cross-entropies.

Name	${\tilde{h}}_{\alpha }\left(f_{1};f_{2}\right)$
**Beta**	$\ln B(a_{2},b_{2})+\frac{1}{\alpha -1}\ln\frac{B(a_{\alpha },b_{\alpha })}{B\left(\alpha \left(a_{1}-1\right)+1,\alpha \left(b_{1}-1\right)+1\right)}$
	$a_{\alpha }:=\alpha a_{1}+\left(1-\alpha \right)a_{2}$, $\;a_{\alpha }>0$
	$b_{\alpha }:=\alpha b_{1}+\left(1-\alpha \right)b_{2}$, $\;b_{\alpha }>0$
$\begin{array}{c} \chi \\ \left(\mathrm{scaled}\right) \end{array}$	$\frac{1}{2}\left(-\ln\frac{2\sigma_{1}^{2}}{\alpha }+k_{2}\ln\sigma_{2}^{2}\sigma_{\alpha }^{2}\right)+\ln\Gamma \left(\frac{k_{2}}{2}\right)$
	$+\frac{1}{1-\alpha }\left(\frac{\alpha k_{1}\ln\frac{\sigma_{\alpha }^{2}\sigma_{1}^{2}}{\alpha }}{2}-\ln\Gamma \left(\frac{k_{\alpha }}{2}\right)+\ln\Gamma \left(\frac{\alpha (k_{1}-1)+1}{2}\right)\right)$
	$\sigma_{\alpha }^{2}:=\frac{\alpha }{\sigma_{1}^{2}}+\frac{1-\alpha }{\sigma_{2}^{2}}$, $\;\sigma_{\alpha }^{2}>0$
	$k_{\alpha }:=\alpha k_{1}+\left(1-\alpha \right)k_{2}$, $\;k_{\alpha }>0$
$\begin{array}{c} \chi \\ \left(\mathrm{non-scaled}\right) \end{array}$	$\frac{-\ln2\alpha }{2}+\ln\Gamma \left(\frac{k_{2}}{2}\right)$
	$+\frac{1}{1-\alpha }\left(-\ln\Gamma \left(\frac{k_{\alpha }}{2}\right)-\frac{\alpha k_{1}\ln\alpha }{2}+\ln\Gamma \left(\frac{\alpha (k_{1}-1)+1}{2}\right)\right)$
	$k_{\alpha }:=\alpha k_{1}+\left(1-\alpha \right)k_{2}$, $\;k_{\alpha }>0$
$\chi^{2}$	$\frac{1}{1-\alpha }\left(-\ln\Gamma \left(\frac{\nu_{\alpha }}{2}\right)+\alpha \ln\Gamma \left(\frac{\nu_{1}}{2}\right)\right)+\ln\Gamma \left(\frac{\nu_{2}}{2}\right)$
	$\nu_{\alpha }:=\alpha \nu_{1}+\left(1-\alpha \right)k$, $\;\nu_{\alpha }>0$
**Exponential**	$\frac{1}{1-\alpha }\ln\frac{\lambda_{1}}{\alpha \lambda_{\alpha }}-\ln\lambda_{2}$
	$\lambda_{\alpha }:=\alpha \lambda_{1}+\left(1-\alpha \right)\lambda_{2}$, $\;\lambda_{\alpha }>0$
**Gamma**	$\ln\Gamma \left(k_{2}\right)+k_{2}\ln\theta_{2}$
	$+\frac{1}{1-\alpha }\left(\ln\frac{\Gamma (k_{1})}{\Gamma (k_{\alpha })}-k_{\alpha }\ln\theta_{\alpha }-\alpha^{2}k_{1}\ln\theta_{1}\right)$
	$\theta_{\alpha }:=\alpha \theta_{1}^{-1}+\left(1-\alpha \right)\theta_{2}^{-1}$, $k_{\alpha }:=\alpha k_{1}+\left(1-\alpha \right)k_{2}$, $\;\theta_{\alpha }>0$
$\begin{array}{c} \mathrm{Gaussian}\\ \left(\mathrm{univariate}\right) \end{array}$	$\frac{1}{2}\left(\ln\left(2\pi \sigma_{2}^{2}\right)+\frac{{\left(\mu_{1}-\mu_{2}\right)}^{2}}{{\left(\sigma^{2}\right)}_{\alpha }}+\frac{1}{1-\alpha }\ln\left(\frac{\alpha \sigma_{2}^{2}}{{\left(\sigma^{2}\right)}_{\alpha }}\right)\right)$
	${\left(\sigma^{2}\right)}_{\alpha }:=\alpha \sigma_{2}^{2}+\left(1-\alpha \right)\sigma_{1}^{2}$, $\;{\left(\sigma^{2}\right)}_{\alpha }>0$
$\begin{array}{c} \mathrm{Gaussian}\\ \left(\mathrm{multivariate}\right) \end{array}$	$\frac{1}{2-2\alpha }\left(-\ln|\alpha |+\ln|A||\Sigma_{1}|+d\right)+\frac{1}{2}\ln\frac{{\left(2\pi \right)}^{n}{\left|\Sigma_{1}\right|}^{2}}{|\Sigma_{2}|}$
	$A:=\alpha \Sigma_{1}^{-1}+\left(1-\alpha \right)\Sigma_{2}^{-1}$, A≻0
	$d:={\left(\mu_{1}-\mu_{2}\right)}^{T}\Sigma_{1}A\Sigma_{2}\left(\mu_{1}-\mu_{2}\right)$
$\begin{array}{c} \mathrm{Gumbel}\\ (\beta_{1}=\beta_{2}=\beta )\; \end{array}$	$\frac{\mu_{2}+\alpha \mu_{1}}{\beta }+\frac{1}{1-\alpha }\left(\ln\frac{\Gamma \left(2-\alpha \right)\eta_{\alpha }}{\alpha \beta }+\frac{\mu_{1}}{\beta }\right)$
	$\eta_{\alpha }:=\alpha e^{-\mu_{1}/\beta }+\left(1-\alpha \right)e^{-\mu_{2}/\beta }$, $\;\eta_{\alpha }>0$
**Half-Normal**	$\frac{1}{2}\left(\ln\left(\frac{\pi \sigma_{2}^{2}}{2}\right)+\frac{1}{1-\alpha }\ln\left(\frac{\alpha \sigma_{2}^{2}}{{\left(\sigma^{2}\right)}_{\alpha }}\right)\right)$
	${\left(\sigma^{2}\right)}_{\alpha }:=\alpha \sigma_{2}^{2}+\left(1-\alpha \right)\sigma_{1}^{2}$, $\;{\left(\sigma^{2}\right)}_{\alpha }>0$
$\begin{array}{c} \mathrm{Laplace}\\ \left(\mu_{1}=\mu_{2}=0\right) \end{array}$	$\frac{\ln b_{\alpha }+\ln\alpha b_{1}}{1-\alpha }+\ln2b_{2}$
	$b_{\alpha }:=\frac{\alpha }{b_{1}}+\frac{1-\alpha }{b_{2}}$, $\;b_{\alpha }>0$
**Maxwell Boltzmann**	$\frac{-\ln2+3\ln\sigma_{2}^{2}}{2}+\ln\frac{\alpha }{\sigma_{1}^{2}}$
	$+\frac{1}{1-\alpha }\left(\frac{3}{2}\ln\frac{\sigma_{\alpha }\sigma_{1}^{2}}{\alpha }-\alpha \ln\frac{\sqrt{\pi }}{2}+\ln\Gamma \left(\alpha +\frac{1}{2}\right)\right)$
	$\sigma_{\alpha }^{2}:=\frac{\alpha }{\sigma_{1}^{2}}+\frac{1-\alpha }{\sigma_{2}^{2}}$, $\;\sigma_{\alpha }^{2}>0$
$\begin{array}{c} \mathrm{Pareto}\\ \left(m_{1}=m_{2}=m\right) \end{array}$	$\frac{1}{1-\alpha }\left(\ln\lambda_{\alpha }-\ln\left(1-\alpha (\lambda_{1}-1)\right)\right)-\ln\lambda_{2}m$
	$\lambda_{\alpha }:=\alpha \lambda_{1}+\left(1-\alpha \right)\lambda_{2}$, $\;\lambda_{\alpha }>0$
**Rayleigh**	$\frac{\ln\sigma_{1}^{2}{\left(\sigma^{2}\right)}_{\alpha }+\ln\alpha +\ln\Gamma \left(\frac{1-\alpha }{2}\right)}{1-\alpha }+\ln2\sigma_{1}^{2}$
	${\left(\sigma^{2}\right)}_{\alpha }:=\alpha \sigma_{1}^{-2}+\frac{1}{2}\ln\frac{2\sigma_{1}^{4}\sigma_{2}^{4}}{\alpha }$, $\;{\left(\sigma^{2}\right)}_{\alpha }>0$

**Table 3:** Differential cross-entropy rates for stationary zero-mean Gaussian sources.

Information Measure	Rate	Constraint
$\begin{array}{c} \mathrm{Shannon}\\ \mathrm{Differential}\\ \mathrm{Cross-Entropy} \end{array}$	$\frac{1}{2}\ln2\pi +\frac{1}{4\pi }\int_{0}^{2\pi }\left[\ln g\left(\lambda \right)+\frac{f(\lambda )}{g(\lambda )}\right]\;d\lambda$	g(λ)>0
$\begin{array}{c} \mathrm{Natural}\;R\text{é}\mathrm{nyi}\\ \mathrm{Differential}\\ \mathrm{Cross-Entropy} \end{array}$	$\frac{1}{2}\ln4\pi^{2}\alpha^{\frac{1}{\alpha -1}}+\frac{1}{4\pi (1-\alpha )}\int_{0}^{2\pi }\ln\frac{j(\lambda )}{{g(\lambda )}^{\alpha }}\;d\lambda$	$\frac{j(\lambda )}{g(\lambda )}>0$
$\begin{array}{c} R\text{é}\mathrm{nyi}\\ \mathrm{Differential}\\ \mathrm{Cross-Entropy} \end{array}$	$\frac{\ln2\pi }{2}+\frac{1}{4\pi (1-\alpha )}\int_{0}^{2\pi }\left[(2-\alpha )\ln g(\lambda )-\ln h(\lambda )\right]d\lambda$	$\frac{g(\lambda )}{h(\lambda )}>0$

**Table 4:** Cross-entropy rates for time-invariant Markov sources.

Information Measure	Rate
$\begin{array}{c} \mathrm{Shannon}\\ \mathrm{Cross-Entropy} \end{array}$	$-\pi_{p}^{T}\left(P⊙\dot{\ln}Q\right)1$
$\begin{array}{c} \mathrm{Natural}\;R\text{é}\mathrm{nyi}\\ \mathrm{Cross-Entropy} \end{array}$	$\frac{1}{\alpha -1}\ln\frac{\lambda (P^{\alpha }⊙Q^{1-\alpha })}{\lambda (P^{\alpha })}$
$\begin{array}{c} R\text{é}\mathrm{nyi}\\ \mathrm{Cross-Entropy} \end{array}$	$\frac{1}{1-\alpha }\ln\lambda \left(P⊙Q^{\alpha -1}\right)$

## Data Availability

Not applicable.

## Appendix A

In Table A1, we describe the distributions of Table 1 and Table 2 (for the multivariate Gaussian, $\mu \in R^{n}$ is a mean vector, and $\Sigma^{2}\in R^{n}\times R^{n}$ is a positive definite covariance matrix).

**Table A1:** Distributions listed in Table 1 and Table 2.

Name	Parameters (Θ)	PDF f(x)	Support
**Beta**	(a>0, b>0)	$B(a,b)x^{a-1}{\left(1-x\right)}^{b-1}$	S=(0,1)
$\begin{array}{c} \chi \\ \left(\mathrm{scaled}\right) \end{array}$	(k>0, σ>0)	$\frac{2^{1-k/2}x^{k-1}e^{-x^{2}/2\sigma^{2}}}{\sigma^{k}\Gamma \left(\frac{k}{2}\right)}$	$S=R^{+}$
$\begin{array}{c} \chi \\ \left(\mathrm{non}-\mathrm{scaled}\right) \end{array}$	(k>0)	$\frac{2^{1-k/2}x^{k-1}e^{-x^{2}/2}}{\Gamma \left(\frac{k}{2}\right)}$	$S=R^{+}$
$\chi^{2}$	(ν>0)	$\frac{1}{2^{\frac{\nu }{2}}\Gamma \left(\frac{\nu }{2}\right)}x^{\frac{\nu }{2}-1}e^{-\frac{x}{2}}$	$S=R^{+}$
**Exponential**	(λ>0)	$\lambda e^{-\lambda x}$	$S=R^{+}$
**Gamma**	(k>0, θ>0)	$\frac{1}{\theta^{k}\Gamma \left(k\right)}x^{k-1}e^{-\frac{k}{\theta }}$	$S=R^{+}$
$\begin{array}{c} \mathrm{Gaussian}\\ \left(\mathrm{univariate}\right) \end{array}$	(μ,$\sigma^{2}>0$)	$\frac{1}{\sqrt{2\pi \sigma^{2}}}e^{-\frac{1}{2}{\left(\frac{x-\mu }{\sigma }\right)}^{2}}$	S=R
$\begin{array}{c} \mathrm{Gaussian}\\ \left(\mathrm{multivariate}\right) \end{array}$	(μ, $\Sigma^{2}$)	$\frac{1}{\sqrt{{\left(2\pi \right)}^{n}\left|\Sigma \right|}}e^{-\frac{1}{2}{\left(x-\mu \right)}^{T}\Sigma^{-1}\left(x-\mu \right)}$	$S=R^{n}$
**Half-Normal**	($\sigma^{2}>0$)	$\sqrt{\frac{2}{\pi \sigma^{2}}}e^{-\frac{1}{2}{\left(\frac{x}{\sigma }\right)}^{2}}$	$S=R^{+}$
**Gumbel**	(μ, β>0)	$\frac{1}{\beta }\exp-\left(\frac{x-\mu }{\beta }+e^{-\frac{x-\mu }{\beta }}\right)$	S=R
**Pareto**	(m>0, a>0)	$am^{a}x^{-(1+m)}$	S=(m,∞)
**Maxwell Boltzmann**	(σ>0)	$\frac{2x^{2}}{\sqrt{\pi \sigma^{6}}}e^{-\frac{1}{2}{\left(\frac{x}{\sigma }\right)}^{2}}$	$S=R^{+}$
**Rayleigh**	($\sigma^{2}>0$)	$\frac{x}{\sigma^{2}}e^{-\frac{1}{2}{\left(\frac{x}{\sigma }\right)}^{2}}$	$S=R^{+}$
**Laplace**	(μ, $b^{2}>0$)	$\frac{1}{2b}e^{-\frac{\left|x-\mu \right|}{b}}$	S=R

**Notes**

- $B\left(a,b\right)=\int_{0}^{1}t^{a-1}{\left(1-t\right)}^{b-1}\;dt$ is the Beta function.
- $\Gamma \left(z\right)=\int_{0}^{\infty }x^{z-1}e^{-x}\;dx$ is the Gamma function.
